# Supplementary material for: Constraint-based modeling identifies new putative targets to fight colistin-resistant A. baumannii infections
Source: Sci Rep. 2017 Jun 16;7:3706. doi: 10.1038/s41598-017-03416-2 (PMC5473915; doi:10.1038/s41598-017-03416-2)
Supplement: Supplementary file 1 — SM1 [file 41598_2017_3416_MOESM1_ESM.zip › SM1.docx]

**Constraint-based modeling identifies new putative targets to fight colistin-resistant *A. baumannii* infections.**

Luana Presta^a^, Emanuele Bosi^a^, Leila Mansouri^a^, Lenie Dijkshoorn^b^, Renato Fani^a^ and Marco Fondi^a*^

a Department of Biology, University of Florence, Florence, Italy

b Department of Infectious Diseases, Leiden University Medical Center, Leiden, The Netherlands

* corresponding author

**Supplementary Materials**

**Supplementary Material S1:**

| **-Supplementary Table 1:** Comparison between Phenotype Microarray experiments and *in silico* outcomes COMPOUND |
| --- |

| COMPOUND | | BIOLOG | | MODEL | RESULT |
| --- | --- | --- | --- | --- | --- |
| Common name | Seed code | Activity Index | Growth | Growth | Agreement |
| 4-Hydroxybenzoic acid | cpd00136 | 3 | 1 | 1 | TP |
| a-D-Glucose | cpd00027 | 2 | 0 | 1 | TN |
| a-Ketobutyric acid | cpd00094 | 4 | 1 | 1 | TP |
| Acetic acid | cpd00029 | 5 | 1 | 1 | TP |
| Acetoacetic acid | cpd00142 | 2 | 0 | 1 | FP |
| Adenosine | cpd00182 | 2 | 0 | 0 | TN |
| Arbutin | cpd03696 | 0 | 0 | 0 | TN |
| Butyric acid | cpd00211 | 5 | 1 | 1 | TP |
| Capric acid | cpd01107 | 0 | 0 | 0 | TN |
| Caproic acid | cpd01113 | 4 | 1 | 1 | FN |
| Citric acid | cpd00137 | 4 | 1 | 1 | TP |
| D-Alanine | cpd00117 | 4 | 1 | 1 | TP |
| D-Fructose | cpd00082 | 0 | 0 | 0 | TN |
| D-Galactose | cpd00709 | 2 | 0 | 0 | TN |
| D-Gluconic acid | cpd00222 | 1 | 0 | 0 | TN |
| D-Glucosamine | cpd00276 | 0 | 0 | 0 | TN |
| D-Malic acid | cpd00386 | 5 | 1 | 1 | TP |
| D-Mannitol | cpd00314 | 0 | 0 | 0 | TN |
| D-Mannose | cpd00138 | 2 | 0 | 0 | TN |
| D-Ribose | cpd00105 | 5 | 1 | 1 | TP |
| D-Serine | cpd00550 | 0 | 0 | 0 | FP |
| D-Sorbitol | cpd00588 | 0 | 0 | 1 | TN |
| D-Tartaric acid | cpd00666 | 1 | 0 | 0 | TN |
| Dihydroxyacetone | cpd00157 | 0 | 0 | 0 | TN |
| Dulcitol | cpd01171 | 0 | 0 | 1 | TN |
| Formic acid | cpd00047 | 0 | 0 | 0 | TN |
| Fumaric acid | cpd00106 | 5 | 1 | 1 | TP |
| Glycerol | cpd00100 | 0 | 0 | 0 | TN |
| Glycine | cpd00033 | 0 | 0 | 0 | TN |
| Glycolic acid | cpd00139 | 0 | 0 | 0 | TN |
| Glyoxylic acid | cpd00040 | 0 | 0 | 0 | TN |
| Inosine | cpd00246 | 0 | 0 | 0 | TN |
| L-Alanine | cpd00035 | 5 | 1 | 1 | TP |
| L-Arabinose | cpd00224 | 5 | 1 | 0 | FN |
| L-Arginine | cpd00051 | 2 | 0 | 0 | FP |
| L-Asparagine | cpd00132 | 5 | 1 | 1 | TP |
| L-Aspartic acid | cpd00041 | 5 | 1 | 1 | TP |
| L-Glutamic acid | cpd00023 | 5 | 1 | 1 | TP |
| L-Glutamine | cpd00053 | 5 | 1 | 1 | TP |
| L-Histidine | cpd00119 | 5 | 1 | 1 | TP |
| L-Homoserine | cpd00227 | 0 | 0 | 0 | TN |
| L-Isoleucine | cpd00322 | 1 | 0 | 0 | TN |
| L-Lactic acid | cpd00159 | 5 | 1 | 1 | TP |
| L-Leucine | cpd00107 | 1 | 0 | 0 | TN |
| L-Lysine | cpd00039 | 0 | 0 | 0 | TN |
| L-Malic acid | cpd00130 | 5 | 1 | 1 | TP |
| L-Methionine | cpd00060 | 0 | 0 | 0 | TN |
| L-Ornithine | cpd00064 | 0 | 0 | 0 | FP |
| L-Phenylalanine | cpd00066 | 5 | 1 | 1 | TP |
| L-Proline | cpd00129 | 5 | 1 | 1 | TP |
| L-Serine | cpd00054 | 0 | 0 | 1 | FP |
| L-Threonine | cpd00161 | 4 | 1 | 1 | TP |
| L-Valine | cpd00156 | 0 | 0 | 0 | TN |
| Malonic acid | cpd00308 | 3 | 1 | 1 | TP |
| Maltose | cpd00179 | 0 | 0 | 0 | TN |
| Oxalic acid | cpd00180 | 0 | 0 | 0 | TN |
| Propionic acid | cpd00141 | 4 | 1 | 1 | TP |
| Putrescine | cpd00118 | 5 | 1 | 0 | FN |
| Pyruvic acid | cpd00020 | 5 | 1 | 1 | TP |
| Quinic acid | cpd00248 | 3 | 1 | 0 | FN |
| Salicin | cpd01030 | 0 | 0 | 0 | TN |
| Succinic acid | cpd00036 | 5 | 1 | 1 | TP |
| Sucrose | cpd00076 | 0 | 0 | 0 | TN |
| Thymidine | cpd00184 | 0 | 0 | 0 | TN |
| Tyramine | cpd00374 | 0 | 0 | 0 | TN |
| Uridine | cpd00249 | 0 | 0 | 0 | TN |

| Abbreviations: | |
| --- | --- |
| TN | True Negative |
| TP | True Positive |
| FN | False Negative |
| FP | False Positive |

**-Supplementary Table 2:** Simmons medium *in silico* composition

| **Simmons medium** | **In silico** | | | |
| --- | --- | --- | --- | --- |
| **Ingredient** | **rxn name** | **compound exchanged** | **lower bound** | **upper bound** |
| Ammonium Dihydrogen Phosphate | EX_cpd00013_e0 | NH3 | -1000 | 1000 |
|  | EX_cpd03811_e0 | NH4(+) | -1000 | 1000 |
|  | EX_cpd00067_e0 | H(+) | -1000 | 1000 |
|  | EX_cpd00009_e0 | HO4P | -1000 | 1000 |
| Sodium Chloride | EX_cpd00971_e0 | Na(+) | -1000 | 1000 |
|  | EX_cpd00099_e0 | Cl(-) | -1000 | 1000 |
| Sodium Citrate | EX_cpd00137_e0 | C6H5O7 | -5 | 1000 |
| Magnesium Sulfate | EX_cpd00254_e0 | Mg2+ | -1000 | 1000 |
|  | EX_cpd00048_e0 | O4S | -1000 | 1000 |
| Bromthymol Blue | missing |  |  |  |
| Agar | missing |  |  |  |
| Dipotassium Phosphate | missing |  |  |  |
| Aerobic condition | EX_cpd00007_e0 | O2 | -5 | 1000 |
| Water | EX_cpd00001_e0 | H2O | -1000 | 1000 |

**-Supplementary Figure 1:** Plot of residual number of reactions after different FBA-bounds setting (various percentage)


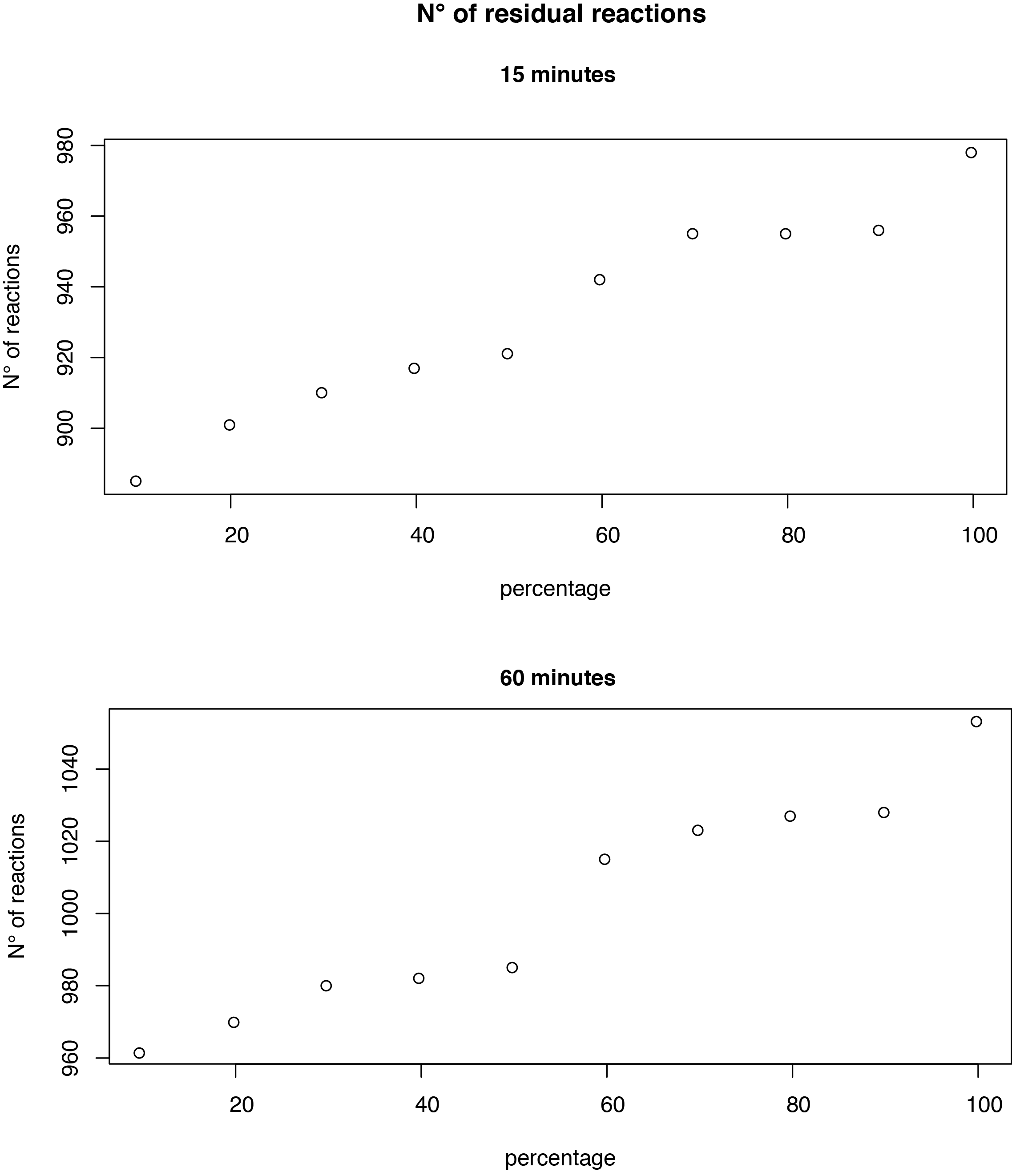


**Supplementary Figure 2:** To account for the influence of the nutrients composition on the number of predicted EGs, 1000 iterations were performed for each simulated condition (Treated 15 min, Treated 60 min, Untreated 15 min and Untreated 60 min) and, for each iteration, new random uptake rates were generated. In other words, for each of these iterations a new virtual environment was created by randomly varying the maximal allowable uptake of each nutrient with respect to the original value (i.e. selecting a random number comprised between 0 and -1000) and by also removing five randomly chosen nutrients from such simulated environment. The number of predicted essential genes was calculated at each iteration. As shown below, the number of essential genes in each condition remains stable throughout all the simulations. Exceptions are represented by those cases in which the changes in the simulated nutrient composition are detrimental for growth (OF flux value = 0); as it might be expected, in these cases, also the number of predicted EGs is equal to 0.


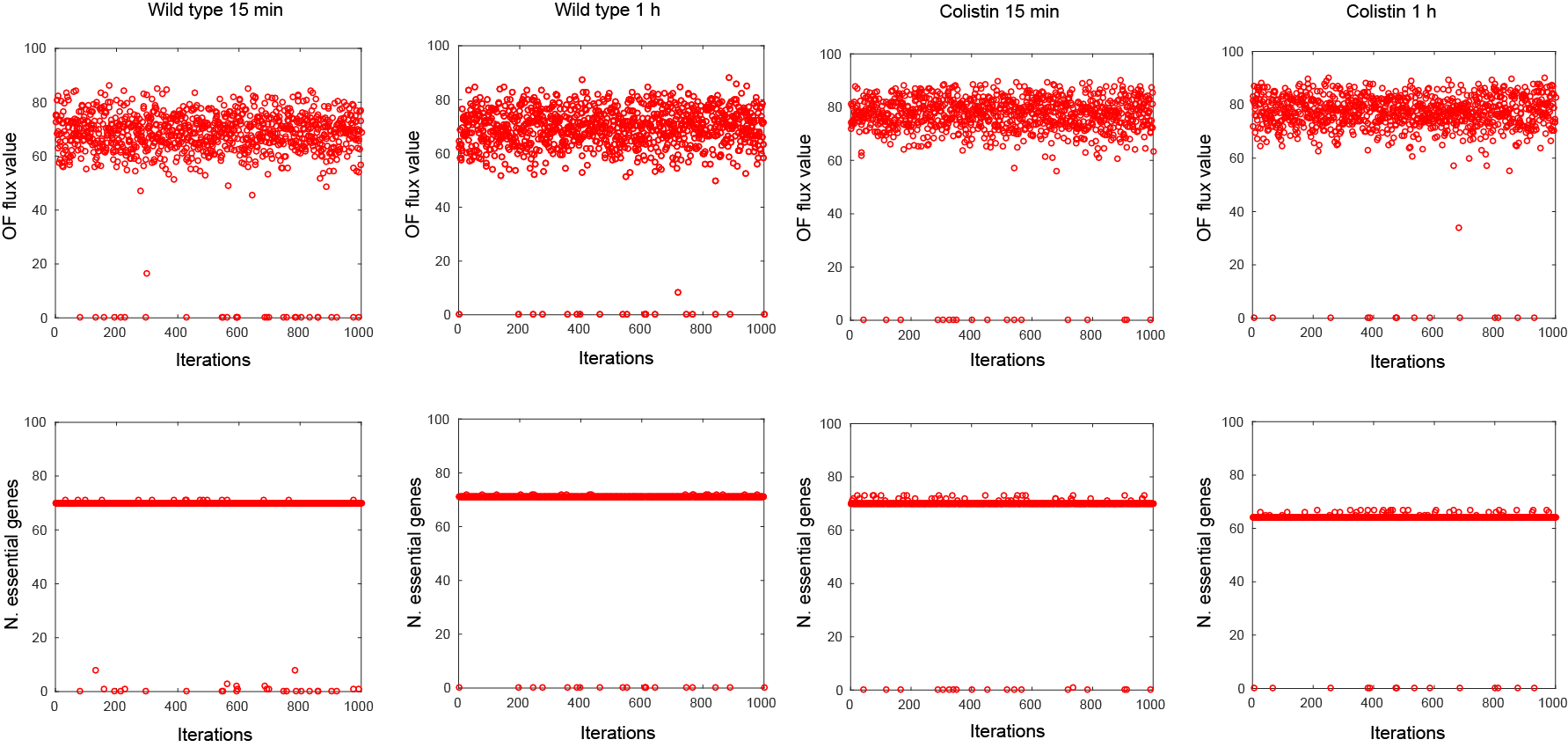


**Supplementary Figure 3:** Boxplot of *A. baumanii* ATCC 19606 EGs distribution among the entire *A. baumannii* species (based on sequence identity percentage)

**Supplementary Material S2:** iLP844’s list of reactions and Gene-Protein-Reaction Rules

**Supplementary Material S3:** iLP844 metabolic model SBML format

**Supplementary Material S4:** EGs set names and functions list in all examined conditions

**Supplementary Material S5:** FVA results and FBA-bounds setting

**Supplementary Material S6:** BLAST output of *A. baumanii* ATCC 19606 model’s EGs against human genome

**Supplementary Material S7:** Commands used to launch the analysis in MATLAB.

**Supplementary Material S8:** Arrays of genes switched-off by MADE in the four conditions.
